# Projected Demographic Trends in the Likelihood of Having or Becoming a Dementia Family Caregiver in the U.S. Through 2060

**DOI:** 10.3390/populations1020010

**Published:** 2025-05-20

**Authors:** Esther M. Friedman, Jessie Wang, Margaret M. Weden, Mary E. Slaughter, Regina A. Shih, Carolyn M. Rutter

**Affiliations:** 1 Institute for Social Research, University of Michigan, Ann Arbor, MI 48104, USA; 2 RAND Corporation, Santa Monica, CA 90401, USA; 3 Rollins School of Public Health, Emory University, Atlanta, GA 30322, USA; 4 Hutchinson Institute for Cancer Outcomes Research, Fred Hutch Cancer Center, Seattle, WA 98109, USA

**Keywords:** caregiving, dementia, disparities, population projections

## Abstract

This study predicts how sociodemographic trends—smaller family sizes, increased longevity, and marital patterns—could affect family care for people with dementia through 2060. By coupling dementia information from the Health and Retirement Study with a well-established kinship microsimulation model, we analyze the impact of demographic changes on the future care landscape, focusing on changes in race and gender differences in two key areas: (1) the availability of family caregivers for people with dementia, and (2) the likelihood of having a family member with dementia, among those without dementia. Our model projections suggest that future dementia cohorts will be more likely to have a living spouse than the current ones, with diminishing gender disparities due to increased male longevity. However, racial disparities will persist, particularly for Black women. The likelihood of older adults lacking spouses, children, and siblings will increase, but remain low. For potential caregivers, we predict an increased likelihood and longer duration of exposure to family members with dementia in future birth cohorts, particularly for Black individuals, potentially placing more people at risk of the adverse health and well-being outcomes associated with caregiving.

## 1. Introduction

As of 2024, approximately 6.9 million Americans ages 65 and older are living with Alzheimer’s disease (AD), a figure anticipated to nearly double by 2060 [1]. AD is marked by progressive cognitive decline, severely limiting an individual’s ability to function independently [2]. The financial burden of AD and related dementias (collectively termed here as “dementia”) is substantial: national estimates suggest that annual dementia-related costs range from USD 159 to 215 billion [3], and these costs are projected to increase by 80% per individual by 2040 [3]. Notably, family care costs, measured in terms of the replacement value if purchased as formal care, constitute about half of the total cost of dementia care [3]. This figure does not account for factors such as diminished emotional well-being, health, and work productivity among family caregivers [4].

The trends in dementia prevalence and incidence have received a fair amount of attention in recent years [5–7], but less focus has been paid to the availability of kin to support them, even though a significant portion of dementia care is provided by family members. The most common type of long-term care involves personal care, including assistance with daily activities, such as bathing, dressing, grooming, toileting, eating, and mobility. Family and friends provide most of this type of support for older adults with dementia [8]. Medicaid serves as the largest payer of formal long-term care, yet eligibility is restricted to individuals with minimal assets. Medicare covers only hospice care and a portion of short-term, post-acute care [9–11]. This limited access to formal services contributes to the reliance on out-of-pocket payments or family-provided care [12]. Furthermore, due to the preference of older adults to remain in their homes and communities [13], caregiving responsibility often falls onto family members.

### 1.1. Differences by Race and Gender

Not all groups are equally likely to experience dementia themselves or have a family member with dementia. For example, the age-specific prevalence of dementia is higher in Black adults than White adults [14,15], and in women than men [6]. When it comes to caregiving, spouses often serve as the primary caregivers for adults with normal cognition, but daughters are more likely to assume this role for adults with dementia [8]. Older women generally have lower spousal availability but greater access to adult children than men, a pattern observed in both non-Hispanic Black and non-Hispanic White populations [16]. Black adults with dementia are more likely to receive assistance from a child or other relatives and less likely to receive support from a spouse than their White counterparts [17]. Understanding this heterogeneity by race and gender is crucial for projecting potential disparities in future trends for different demographic subgroups.

### 1.2. Potential Demographic Drivers

The demographic drivers of the trends in dementia care have been identified as an important, albeit under-examined, factor for understanding later-life family caregiving [18]. One issue pertaining to the availability of family care is shrinking family sizes, which have been declining steadily since the 1960’s [19], and could mean fewer family caregivers will be available in the future. While care for older adults is most often provided by one family member [20–23], usually the wife or daughter, the person primarily responsible for care within a family changes over time [24]. For dementia care in particular, multiple family members may share caregiving tasks [25]. Smaller family sizes do not necessarily reduce the availability of care, but could leave fewer family caregivers per recipient [26].

Other recent sociodemographic changes may also affect the supply of caregivers in the future. The higher rates of divorce among the cohorts now reaching older ages may mean that fewer older adults have the support of a spouse, and more older parents, especially fathers, have estranged relationships with their adult children [27,28]. On the other hand, blended and step-families may increase the kin available to help older parents, though evidence suggests that the children of divorce are less likely to provide care to step-parents [28]. Increasing longevity, especially for men [29], means that spouses may have more healthy years available to provide care to their partners. These diverging trends make it difficult to assess the extent to which family care for dementia will be available in the future, and who will be available to provide this care. A family perspective is crucial to understanding the implications of demographic trends for both the older adults who need care and the relatives who may be called upon to provide it.

### 1.3. Microsimulation Models and Prior Work on Family Care Projections

Microsimulation models build on existing evidence to project population-level outcomes under different assumptions. They are thus an ideal method for simultaneously projecting the availability of family care among people with dementia and, among those without dementia, their exposure to kin with dementia—although they are not typically used in this manner. The ‘usual’ simulation model uses a unit of analysis that is the total U.S. population and estimates from the microdata about individuals (i.e., population microsimulation models). In contrast, in a ‘family’ projection model the unit of analysis is the individual, and their families are also simulated and tracked over time. The ‘usual’ models project demand factors, such as the prevalence or size of the AD population [30–36], and can even explore supply (e.g., formal care workforce) [37]. A model of kin networks that simulates individuals and their family relationships, however, is necessary to answer questions about the changing constraints on who is available for family-based caregiving.

Only a handful of studies have examined the availability of family care by simulating family networks [38–41], either through microsimulation models or a matrix population modeling approach [42]. These have mostly focused on a limited definition of family and have not distinguished among different types of family members. They have also primarily focused on the availability of family members to older adults and have paid little, if any, attention to the broader population of potential caregivers. Finally, the prior studies, with one notable exception [42], have not explored patterns of care for individuals with dementia despite the fact that dementia caregivers provide more hours of care, and experience worse health consequences compared to caregivers for other health conditions.

### 1.4. Current Study

The current study considers questions at the intersection of demographic research—the projected size and sociodemographic composition of extended families in the US—and epidemiological research—the projected size and sociodemographic composition of the US population with dementia. Specifically, we explore the following research questions.

1. What are the population dynamics influencing the availability of family care to U.S. adults ages 65 and over with dementia?
  - What proportion of individuals with dementia are expected to have no surviving spouse, sibling, or children who could provide care?
  - How will this change with time?
  - How is this expected to differ by race and gender?
2. What are the population dynamics influencing the likelihood of being a potential caregiver to someone with dementia among the U.S. population ages 15 and over without dementia?
  - What proportion of individuals ages 15 and over will be a potential caregiver to a family member with dementia?
  - How will this change with time?
  - How is this expected to differ by race and gender?
  - Who among potential caregivers’ family members will be most likely to develop dementia (e.g., grandparents, parents, spouse, or siblings)?

To answer these questions, we use an open-source population microsimulation model that has been employed and refined through over a half-century of demographic research [41,43,44] and has been recently expanded to include information on probable dementia status [45].

## 2. Materials and Methods

### 2.1. Data and Modeling

The data include simulated and projected microdata for the U.S. population that describe the life history (e.g., birth, unions, childbirth, and death) and development of dementia for White men and women and Black men and women. To project the future family structure and availability of caregivers, we used Socsim, a validated open-source demographic microsimulation program developed in the 1970s [43,44]. Socsim takes as inputs an initial population and demographic rates and returns an updated population (up to age 100), including kinship networks (parents, children, siblings, aunts, and uncles). Every month, each individual faces the risk of a number of events occurring, including childbirth, death, and marriage. This results in a simulated population that mirrors the U.S. population, including each individual’s extended kin, which can be used to calculate both exposure to and availability for dementia caregiving.

We built on previously reported and validated applications of Socsim to examine trends in kin networks [41] and differences in grandparenthood by race and sex [46] that models the Black and White populations (but not other races or specific ethnicities) from 1880 to 2013 based on census data, and projects population trends to 2060. Consistent with prior work, we initialized Socsim based on 1880 census data, and simulated Black and White populations separately to allow for differences by race. Current-day and future populations were then generated by simulating this initial population forward, using annual age–sex–race-specific and age–race–parity-specific transition rates estimated from harmonized compendia of observed and projected data inputs from the U.S. Census Bureau. These inputs were annual observed and projected life expectancy; Total Fertility Rate (TFR); male birth proportions; marital status and parity status birth proportions; and marriage, remarriage, divorce, and partnership rates. We compared model projections to vital registry statistics to ensure the model adequately captured TFR and life expectancy.

We made two modifications to the Socsim model used in prior work. First, because our focus is on availability of family caregivers, we expanded simulated parity from 3 levels (0, 1, or 2 or more prior births) to 5 levels (0, 1, 2, 3, or 4 or more prior births). In addition, given our focus on dementia care, we augmented the simulated Socsim population by applying a model for dementia incidence to predict dementia onset based on data from the Health and Retirement Study [47] and published estimates of age- and race-specific hazard ratios for death after dementia diagnosis, as described in detail elsewhere [45]. This approach simulates dementia onset and death after dementia onset, and is consistent with observed dementia prevalence rates [48] and post-diagnosis survival time [49], without changing the age of death assigned by Socsim.

Microsimulation models, such as this one, are best suited for examining within-population differences, such as, differences between groups, and changes in trends over time. Thus, we focused on the combined influence of demographic trends on the caregiving context over time, across birth cohorts, and among population subgroups (i.e., race and gender).

### 2.2. Analytic Approach

To examine changes over time in family availability to people with dementia ages 65 and over by race and gender, we compared the projected population of adults ages 65 to 100 with dementia for two time periods: 2020–2024 and 2055–2059. We chose these periods to capture changes between a contemporary and a future time period, so as to assess how predicted family availability will change over time, and how it will look by the end of the simulation. We examined changes in the percent of spouses, children, and siblings, the primary family caregivers to people with dementia.

For our analyses of changes over time for exposure to family members with dementia among potential caregivers, we explored exposure to close kin with dementia among Black and White individuals ages 15–90 without dementia themselves (over their “at risk” lifetime). While we cannot tell whether individuals actually provide care, we consider these individuals to be in the risk pool for becoming a caregiver or, simply, potential caregivers. We limit analyses to age 90 because we projected the 1970 birth cohort out 90 years (to 2060). While caregivers may be younger than age 15 or older than 90, this should capture prime caregiving ages. We include “youth caregivers” under age 18 because there is increasing evidence that such young adults assist with caregiving for family members, particularly in non-White families. One study estimated that over 9% of youth aged 15–18 (roughly 1.6 million individuals) provide care to a family member [50]. We examined lifetime exposure to someone with dementia, how it is projected to change across the two cohorts, intensity of exposure (i.e., exposure to multiple individuals with dementia), and to whom potential caregivers are exposed (i.e., grandparents, parents, spouses, and siblings).

### 2.3. Kinship Measures

There are many ways to think about the family members who are available to provide care to people with dementia. We focused on the family relationships that most commonly undertake caregiving roles: spouses, children, siblings, and grandchildren. For the analyses of family caregiver availability to people with dementia, we counted the projected number of kin types over time and explored changes in the distribution of kin types over time. For the analyses of lifetime likelihood of being a family caregiver, we counted the projected number and type of relatives with dementia (i.e., spouse, biological parent, grandparent, and sibling), and the total months of exposure to these relatives among people without dementia aged 15 to 90. Full- and half- siblings and children were included in the life tables, but step-relatives were not.

## 3. Results

### 3.1. Changes over Time in Family Caregiver Availability to People with Dementia, by Race and Gender

In Table 1, we examine the prevalence of not having family members to provide care according to a widening definition of the missing family members, i.e., ‘no living spouse’—including both marital and cohabiting partners (first panel); ‘no living spouse or child’ (second panel); and ‘no living spouse, child, or sibling’ (third panel).

We first consider, in the left-most panel of columns, the likelihood that an older adult with dementia is living without a spouse due to being widowed, divorced, or never married. For the period 2020–2024, we project that upwards of two-thirds of women had no surviving spouse (i.e., 69.8% for White women and 78.0% for Black women), while for men the rate was no higher than about half of that (i.e., 37.1% for White men and 51.9% for Black men). In contrast, for the 2050–2059 period, nearly 32% of White men, almost 50% of Black men, about 55% of White women, and almost 66% of Black women will have no spouse.

These rates indicate an improvement over time for all groups—all four groups are less likely to have no spouse in the 2050–2059 period compared to 2020–2024. This is especially true for women. In both periods, the largest disparity is seen for Black men compared to White men. For both time periods, Black men are more likely to have no surviving spouse than are their White counterparts. Black women are the most disadvantaged relative to the other groups for both periods.

The next set of columns in Table 1 shows the combined lack of two types of kin crucial for providing dementia care, spouses and children. Far fewer people lack access to both a spouse and children than those lacking a spouse, with rates for the former below 10% for 2020–2024 for all the groups but Black women. These rates are projected to increase about three percentage points for White men and White women by 2055–2059 (i.e., to 10.1% and 11.8%, respectively), and by about four percentage points for Black men and Black women (i.e., to 13.7% and 17.3%, respectively).

The final set of columns in Table 1 shows the fraction of people without spouses, children, or siblings, an even smaller percentage of the population than seen in the previous panels. The rates, in general, are higher for women than men, reflecting the gender difference in life expectancy. The projected rates for 2055–2059 suggest that more people will lack these types of family members, with the exception of Black women. This growth is small in absolute value (1.5 percentage points for White men and less than 1 percentage point for Black men and White women), but significant relative to the base values from 2020 to 2024. For example, White men will experience the greatest decline (75% decline in having a spouse, child, or sibling caregiver relative to their initial rates of availability). The exception of Black women indicates that the Black sibling group trends differently from the White sibling group.

In results not shown here, we also examined kinship with the inclusion of grandchildren, but adding grandchildren showed very similar percentages to those of the third panel, suggesting that only a very small fraction of the population has no spouse, no children, and no siblings, but has living grandchildren. We also conducted a sensitivity test that included both biological and step-relationships, and the results were consistent with those shown here.

### 3.2. Changes over Time in Exposure to Family Members with Dementia Among Potential Caregivers, by Race and Gender

**Figure 1.**
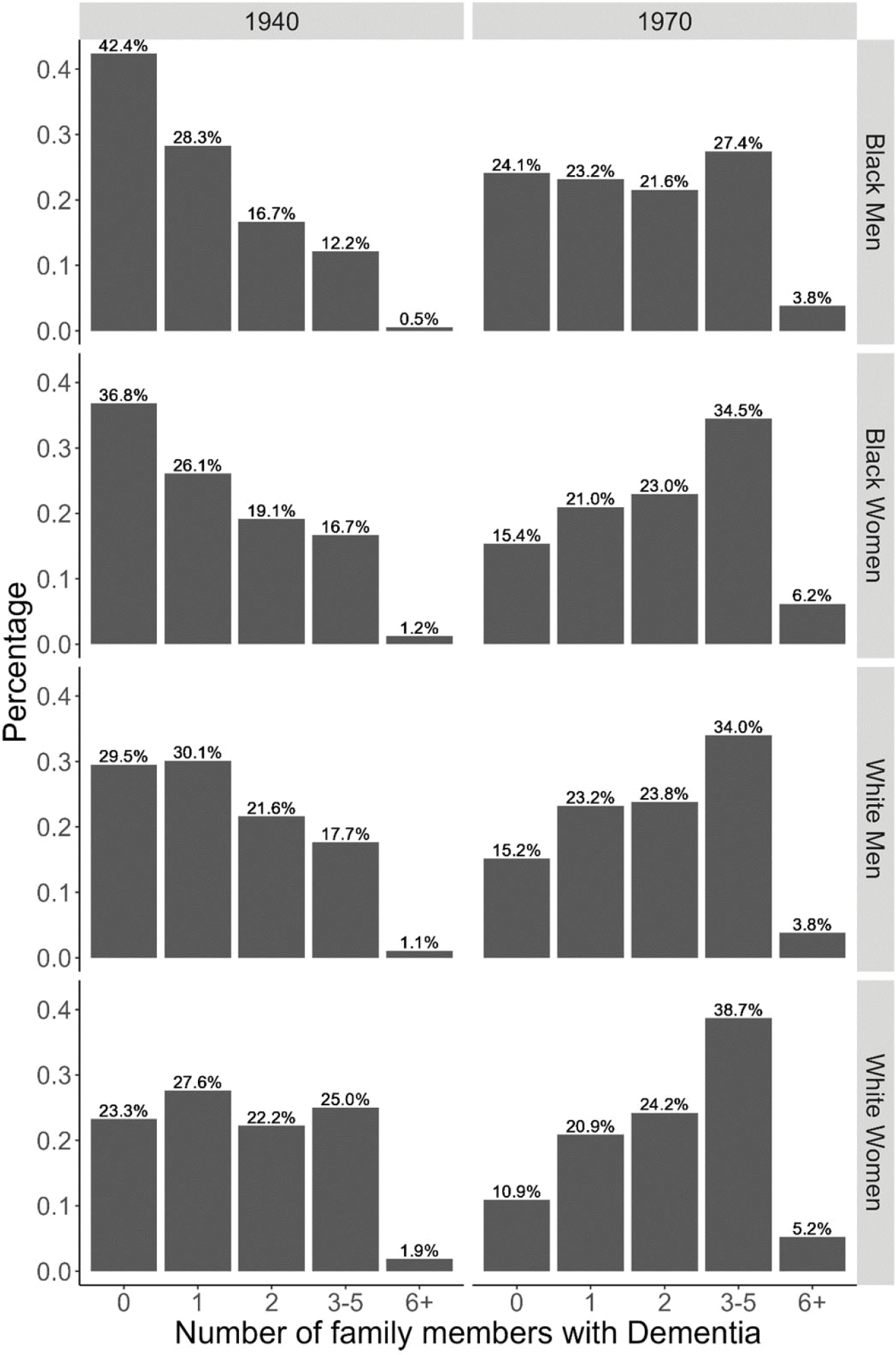
Number of family members living with dementia during person’s lifetime, by gender, race, and birth cohort.

In the second set of analyses, we examine the likelihood of having a close family member with dementia among the population of potential caregivers. We compare the likelihood between the 1940 and 1970 birth cohorts. We start by counting the number of relatives (including grandparents, parents, siblings, and spouse) with dementia an individual could have exposure to over their lifetime, presented in Figure 1.

Figure 1 shows that, for Black men and women in the 1940 birth cohort, 42.4% and 36.8%, respectively, lived without ever being exposed to close kin with dementia. There is also a sizable group of White men and women in this cohort who were never exposed to close kin with dementia (29.5% and 23.3%, respectively). The distributions move entirely to the right for all race and gender subpopulations in the 1970 birth cohort. The size of the group with no relatives living with dementia is more than halved for females (from 36.8% to 15.4% for Black women and from 23.3% to 10.9% for White women), and almost halved for males (from 42.4% to 24.1% for Black men and from 29.5% to 15.2% for White men). A larger proportion of people in the 1970 birth cohort will have exposure to at least one close family member with dementia, and they will also be exposed to more relatives with dementia, than the 1940 cohort. Of people who had or will have exposure to at least one relative with dementia, most people in the 1940 cohort had one or two relatives with dementia (with the exception of White women), whereas most people in the 1970 cohort are projected to have exposure to 3–5 such relatives.

**Figure 2.**
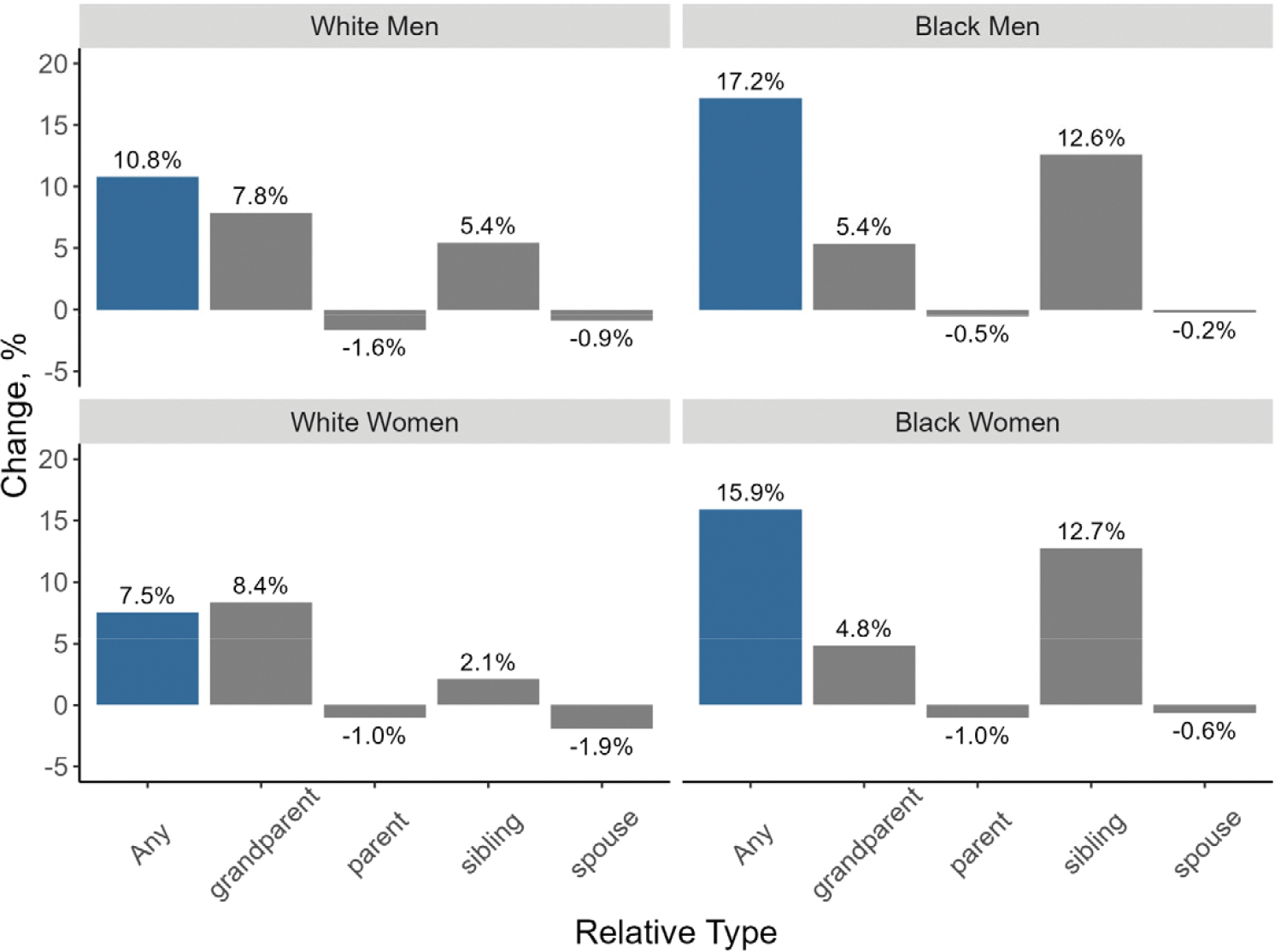
Differences in the percent of the population with different types of relatives with dementia between 1940 and 1970 birth cohorts.

In Figure 2, we explore the changes in exposure to different relatives with dementia for the 1940 and 1970 birth cohorts by race and gender. The changes in exposure to parents and spouses with dementia between the 1940 and 1970 birth cohorts are negligible. Where we see notable differences is in the grandparent and sibling relationships. All the groups show a sizable increase in their risk of potentially providing care to grandparents with dementia, though this is higher for White (7.8% for men and 8.4% for women) than for Black (5.4% for men and 4.8% for women) individuals. It is notable that the likelihood of having siblings with dementia increases significantly more for Black (12.6% increase for men and 12.7% for women) than for White (5.4% for men and 2.1% for women) individuals between the two cohorts.

To identify the differences between the two cohorts in the amount of time spent “at risk” of becoming a care provider close kin, in Table 2, we present the months exposed to family members with dementia as a percentage of the lifetime at-risk months. The at-risk months are the total number of months a group is eligible to be caregivers (i.e., age 15–90 with no dementia themselves), and the exposed months are the total number of months a group has at least one relative (i.e., grandparents, parents, siblings, or spouse) with dementia. The 1940 cohort has exposure rates between 12% and 13% for all the groups, and the 1970 cohort rates are 3% to 4% higher. For both cohorts, Black individuals spend slightly more of their lives at risk of becoming a caregiver to someone with dementia than do White individuals, and the difference is more pronounced for the 1970 cohort (16.2% and 16.3% for Black men and women compared to 15.4% and 15% for White men and women) than the 1940 cohort. We also show the percentage increase in the total number of months exposed in Table 2. Black women see the highest increase between the 1940 and 1970 birth cohorts in months exposed (230%), followed by slightly lower rates for Black (225.2%) and White men (224.2%), and the lowest rate for White women (177.4%).

To capture the intensity of the exposure to caregiving needs, we also calculate the percent of exposed months in which two or more relatives have dementia in the last two columns of Table 2. For the 1940 cohort, the exposure to more relatives with dementia is higher for men (5.2% for White men and 5.6% for Black men) than for women (4.5% for White women and 4.3% for Black women). This pattern remains for the 1970 cohort, and all the subgroups experience a moderate increase of 0.5 to 1.1 percentage points.

**Figure 3.**
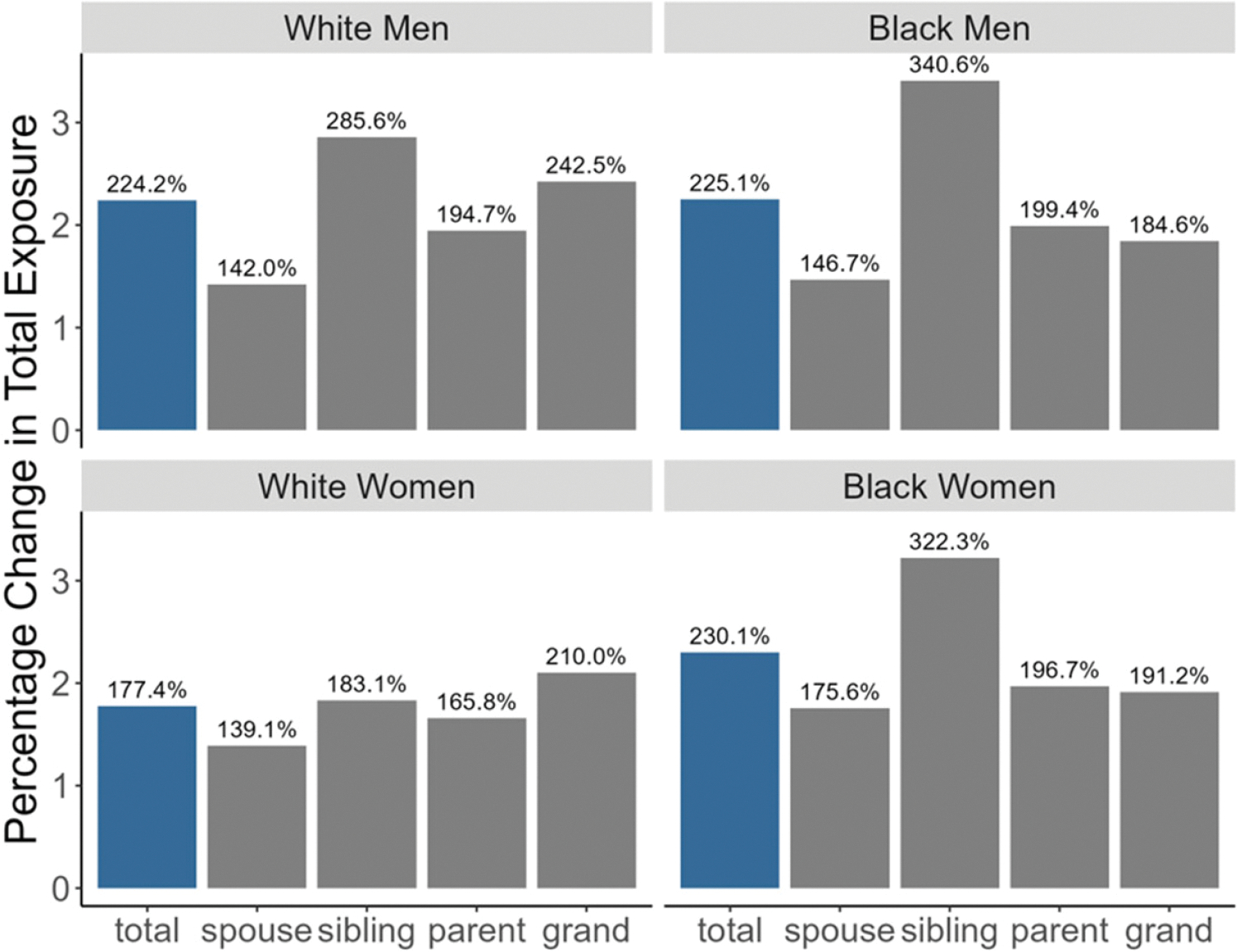
Percentage change in total months exposed to caregiving risk by relative type between 1940 and 1970 birth cohorts.

To show the sources of the increase in exposure, we graph the percentage differences in the total months exposed to a family member with dementia, by relative types and by race and gender, in Figure 3. We report the percent changes here, but the fraction of the population in each category by race and gender are displayed in Supplemental Figure S1. In Figure 3, we see the differences by race, regarding how many family members with dementia individuals are likely to be exposed to, and how this changes by cohort. These racial differences across the two cohorts come from all types of relatives, with an especially noticeable surge from siblings. Notably, compared to those born in 1940, Black men born in 1970 are projected to experience a 340.6% increase in their siblings who will have dementia. White men will also see a significant increase from their siblings (285.6%). Meanwhile, White men and women will see a larger increase (242.5% and 210%) in exposure from grandparents than Black men and women (184.6% and 191.2%).

## 4. Discussion

As the baby boomer generation ages, there are growing concerns as to whether older adults, particularly those with care needs due to dementia, will have sufficient familial support available from spouses, children, and other relatives. Our findings reveal distinct disparities in the likelihood of having no surviving spouse, with women generally disadvantaged compared to men. However, the projections suggest that older adults will increasingly have a living spouse in the future, with women—currently more likely to lack this close kinship—experiencing more significant changes over time, bringing them closer to parity with men. The incidence of having neither a spouse nor a biological child is low, but is projected to increase among all racial and gender groups.

Black women are predicted to remain at a disadvantage with regard to caregiver availability in the future, with the highest rates of no available spouse. When we take a more inclusive view of family caregiver availability, it is rare for someone to be without a spouse and child and rarer still to lack a spouse, child, and siblings, with race and gender differences becoming less pronounced. This underscores the resilience of family networks, even amid demographic shifts.

Our subsequent set of analyses shifts the focus to the population at risk of becoming a caregiver to someone with dementia; that is, to the potential caregivers. Among the 1940 birth cohort, 36.8% of Black women and 42.4% of Black men never encountered a close relative with dementia. Moreover, a substantial proportion of White men and women—29.5% and 23.3%, respectively—in this cohort also had no exposure to close kin with dementia. In contrast, the 1970 birth cohort demonstrates a marked increase in exposure to relatives with dementia, with more months exposed and a higher likelihood of exposure to multiple family members with dementia.

Consistent with other works [42], we find that Black individuals have more family members with dementia compared to their White counterparts (especially White women), potentially placing them at greater risk of being a caregiver. We also find that, across both cohorts, Black individuals experience a greater proportion of their lives exposed to caregiving risk than White individuals, a disparity that becomes even more prominent in the 1970 cohort. White women have the lowest rates of potential caregiving compared to all other race and gender groups. We also show that more recent birth cohorts may be more likely to face caregiving for multiple family members with dementia at the same time. Indeed, the time trends from the Caregiving in the U.S. study from 2015 to 2020 show an increase from 18% to 24% of family caregivers caring for more than one person [51].

Racial variations also manifest in the types of family members with dementia that individuals are likely to have and how these exposure patterns evolve over time. There is a particularly dramatic escalation over time in the likelihood of having a sibling with dementia among Black individuals. Conversely, White men and women face a greater increase in exposure from grandparents compared to their Black counterparts.

This is one of the first studies to examine the future caregiving landscape from the perspectives of both older adults with dementia in need of care and the family members likely to care for them. Only one other study has projected kin availability for people with dementia through 2060 [42]. Our study complements this work in our use of historic data and microsimulation models to investigate the changes in family caregiver availability across the two periods we investigate, as well as in our thorough exploration of the likelihood of having family members with dementia. We do this by examining the differences between two birth cohorts regarding their length of time exposed to someone with dementia, how many potential relatives with dementia each gender and racial subgroup are exposed to, and how exposure to different family members changes over time. The current study expands our understanding not only of whether family caregiving is available, but who is likely to become a family caregiver.

These findings carry substantial implications for policy development aimed at assisting older adults. Projecting the availability of family caregivers in the future is important for planning for future demand for Medicaid and the expansion of formal long-term care services. In addition, any increased burden on individual caregivers could have notable consequences for population health in the future, as caregivers often experience adverse physical and psychological health compared to non-caregivers [52–54], and the physical and psychological toll could be even more detrimental in the future if caregiving demands are longer-lasting or more intensive, as suggested by the increase in exposure to multiple family members with dementia and a longer duration of exposure. Furthermore, caregiving obligations for family could affect employment and financial stability for a large subset of the population, even over a longer term [55,56]. This could also have implications for family dynamics in the future, as caregiving for someone with dementia can strain familial relationships and may even have spillover effects on caregivers’ relationships with their own children [57,58]. Our simulations suggest that Black men and women will experience greater and more prolonged exposure to family members with dementia in their lifetimes, which could place them at greater risk of being a caregiver than White individuals.

While our work provides important insights into the future of family care, there are a few limitations to our methods that call for future work. First, the results of these models reflect the kinship of single-race, native-born, non-Hispanic Black and White individuals. The absence of multiracial individuals and families in the projections therefore limits the generalizability of our findings, and results in a potentially lower predictive power for farther in the future, when the population will be more diverse. The absence of migration limits the generalizability of these models, but has important advantages for this work, as the “closed system” allows us to reconstruct the entire kinship network of every simulated individual and determine the demographic characteristics of their kin at any time point. Nonetheless, because these models do not include migration and are constrained by the parameters of Socsim, the specific values will not necessarily be identical to real-world estimates. These models are most useful for examining within-population differences. We therefore focus our results on comparisons of trends—those across race and gender groups and over time.

These models estimate kinship networks, but care can come from friends, neighbors, more distant relatives, and other unrelated individuals. They are an important part of caregiving networks [59], but beyond the scope of this investigation. We primarily focus on spouses and biological family in these analyses, as there is evidence that they are the ones most likely to provide care, but future work could explore step-relatives. We identify individuals as potential caregivers if they have someone with dementia in their family and do not have dementia themselves; however, there are many factors that place some people at greater risk of being a caregiver than others. In particular, those in ill health may be physically unable to assist with caregiving. While beyond the scope of this study, future work could incorporate more information on the sociodemographic characteristics of individuals into these models to develop a more precise estimate of the caregiving risk pool. In our analyses of caregiver availability for people with dementia, we focus on adults ages 65 and older, the population most likely to have a dementia diagnosis, but do not examine younger-onset dementia. In addition, our models do not account for COVID-19 related changes to mortality because we build on models that were developed before the pandemic, but this will be an important addition for future modeling work. While we focus on gender and racial differences, we do not examine economic differences. Future research could augment these models to examine how family economic situations could result in reliance on and the demand for publicly and privately funded care.

This work is an important step in projecting the future of dementia care from both the care recipient and caregiver perspectives, and for identifying anticipated disparities in these trends. Our findings suggest that Black women will remain at a disadvantage relative to other racial-by-gender groups regarding spousal caregiver availability in the future. In addition, our projections indicate that everyone, and especially Black individuals, will be at increased risk in the future of having someone with dementia in their family and will experience more months of exposure to someone with dementia. Taken together, this suggests that culturally competent caregiver support programs (e.g., training, counseling, and respite care services) and services supporting older adults with dementia may be needed to an even greater extent in the future, and should be adapted for the populations at greatest risk of needing this support. Programs like these will be critical for ensuring that current and future family caregivers and older adults with dementia have access to the services they need to maintain their physical, psychological, and financial well-being for decades to come.

## Supplementary Material

Supplementary Materials

**Supplementary Materials:** The following supporting information can be downloaded at: https://www.mdpi.com/article/10.3390/populations1020010/s1, Figure S1: Fraction of the population from the 1940 and 1970 birth cohorts with relatives living with dementia by relative type, race, and gender.

## Figures and Tables

**Table 1.** Projected percentages of U.S. adults with dementia without potential family caregivers.

	No Living Spouse (%)			No Living Spouse or Children (%)			No Living Spouse, Children, or Siblings (%)		
	
	2020–2024	2055–2059	Change	2020–2024	2055–2059	Change	2020–2024	2055–2059	Change

White Men	37.1	31.8	−5.3	7.0	10.1	3.1	2.0	3.5	1.5
Black Men	51.9	49.7	−2.2	9.8	13.7	4.1	2.5	3.4	0.9
White Women	69.8	54.5	−15.3	8.5	11.8	3.3	3.2	4.1	0.9
Black Women	78.0	65.8	−12.2	12.8	17.3	4.5	4.7	4.3	−0.4

Data source: Percentages are estimated from Socsim simulation and projection of dementia and kinship conducted separately for Black and White U.S. adults ages 65+. Simulation is conducted in months and data are grouped in 5-year increments. Children and siblings include biological relationships only. Change values that are positive indicate an increase over time in having no family members of that type.

**Table 2.** Percent exposed, change in duration of exposure, and intensity of exposure to family members with dementia in the 1940 and 1970 birth cohorts, for population ages 15–90 without dementia.

	% Exposed		% Change in Duration of Lifetime Exposure (in Months)	% Exposed to 2+ Family Members	
	
	1940	1970	1940–1970	1940	1970

White Men	12.2	15.4	224.2	5.2	6.3
Black Men	12.8	16.2	225.1	5.6	6.4
White Women	12.2	15.0	177.4	4.5	5.0
Black Women	12.3	16.3	230.0	4.3	5.1

Notes: Percentages are estimated from Socsim simulation and projection of dementia and kinship conducted separately for the Black and White U.S. population. By group, the total number of months the 1940 birth cohort is eligible to be caregivers (i.e., age 15–90 with no dementia themselves) are as follows: 1,331,539 (White men), 1,719,357 (Black men), 1,634,927 (White women), and 2,268,988 (Black women). For the 1970 birth cohort, they are as follows: 3,423,541 (White men), 4,417,219 (Black men), 3,697,640 (White women), and 5,659,768 (Black women). Exposed months are the total months an individual is eligible to be a caregiver and has at least one close family member (spouse, parent, or grandparent) with dementia. Exposure intensity is measured by the percentage of exposed months during which one has two or more close kin with dementia.

## Data Availability

The data used in the Socsim models came from the census and the Health and Retirement Study. All the data are publicly available.

## References

[1] Alzheimer’s Association. Alzheimer’s Disease Facts and Figures: Special Report Mapping a Better Future for Dementia Care Navigation. Alzheimers Dement. 2024, 19, 1598–1695. Available online: www.alz.org/getmedia/a7390c29-e4c0-4d3c-b000-377b6c112eea/alzheimers-facts-and-figures-special-report.pdf (accessed on 4 February 2025).

[2] American Psychiatric Association. Diagnostic and Statistical Manual of Mental Disorder, 5th ed.; American Psychiatric Publishing: Washington, DC, USA, 2013.

[3] Hurd MD; Martorell P; Delavande A; Mullen KJ; Langa KM Monetary Costs of Dementia in the United States. N. Engl. J. Med. 2013, 368, 1326–1334. 23550670 10.1056/NEJMsa1204629 PMC3959992

[4] Reinhard SC; Kassner E; Houser A; Mollica R Raising Expectations: A State Scorecard on Long-Term Services and Supports for Older Adults, People with Physical Disabilities, and Family Caregivers. 2011. Available online: https://ltsschoices.aarp.org/scorecard-report/2014/raising-expectations-2014 (accessed on 4 February 2025).

[5] Hudomiet P; Hurd MD; Rohwedder S Dementia Prevalence in the United States in 2000 and 2012: Estimates Based on a Nationally Representative Study. J. Gerontol. Ser. B. 2018, 73, S10–S19. 10.1093/geronb/gbx169 PMC6018928 29669104

[6] Hudomiet P; Hurd MD; Rohwedder S Trends in Inequalities in the Prevalence of Dementia in the United States. Proc. Natl. Acad. Sci. USA 2022, 119, e2212205119. 36343247 10.1073/pnas.2212205119 PMC9674270

[7] Mukadam N; Wolters FJ; Walsh S; Wallace L; Brayne C; Matthews FE; Sacuiu S; Skoog I; Seshadri S; Beiser A; Changes in Prevalence and Incidence of Dementia and Risk Factors for Dementia: An Analysis from Cohort Studies. Lancet Public Health 2024, 9, e443–e460. 38942556 10.1016/S2468-2667(24)00120-8

[8] Friedman EM; Shih RA; Langa KM; Hurd MD US Prevalence and Predictors of Informal Caregiving for Dementia. Health Aff. 2015, 34, 1637–1641. 10.1377/hlthaff.2015.0510 PMC4872631 26438738

[9] Harris-Kojetin LD; Sengupta M; Park-Lee E; Valverde R Long-Term Care Services in the United States: 2013 Overview; National Center for Health Statistics: 2013. Available online: www.cdc.gov/nchs/data/nsltcp/long_term_care_services_2013.pdf (accessed on 4 February 2025). 26158640

[10] Redfoot D; Feinberg L; Houser AN The Aging of the Baby Boom and the Growing Care Gap: A Look at Future Declines in the Availability of Family Caregivers; AARP Public Policy Institute: Washington, DC, USA, 2013. Available online: https://www.caregiver.org/uploads/2023/02/baby-boom-and-the-growing-care-gap-insight-AARP-ppi-ltc.pdf (accessed on 4 February 2025).

[11] SCAN Foundation. Who Pays for Long-Term Care in the U.S.? SCAN Foundation: Long Beach, CA, USA, 2013. Available online: https://www.thescanfoundation.org/sites/default/files/who_pays_for_ltc_us_jan_2013_fs.pdf (accessed on 4 February 2025).

[12] Bookman A; Kimbrel D Families and Elder Care in the Twenty-First Century. Future Child 2011, 21, 117–140. 22013631 10.1353/foc.2011.0018

[13] Johnson RW; Wiener JM A Profile of Frail Older Americans and Their Caregivers; Urban Institute: Washington, DC, USA, 2006. Available online: https://www.urban.org/sites/default/files/publication/42946/311284-A-Profile-of-Frail-Older-Americans-and-Their-Caregivers.PDF (accessed on 4 February 2025).

[14] Matthews KA; Xu W; Gaglioti AH; Holt JB; Croft JB; Mack D; McGuire LC Racial and Ethnic Estimates of Alzheimer’s Disease and Related Dementias in the United States (2015–2060) in Adults Aged ≥ 65 years. Alzheimers Dement. 2019, 15, 17–24. 30243772 10.1016/j.jalz.2018.06.3063 PMC6333531

[15] Zhu Y; Chen Y; Crimmins EM; Zissimopoulos JM Sex, Race, and Age Differences in Prevalence of Dementia in Medicare Claims and Survey Data. J. Gerontol. Ser. B 2021, 76, 596–606. 10.1093/geronb/gbaa083 PMC7887731 32588052

[16] Choi H; Heisler M; Norton EC; Langa KM; Cho T-C; Connell CM Family Care Availability and Implications for Informal and Formal Care Used by Adults with Dementia in the US: Study examines family care availability and implications for informal and form care used by adults with Dementia in the United States. Health Aff. 2021, 40, 1359–1367. 10.1377/hlthaff.2021.00280 PMC8647567 34495713

[17] Parker LJ; Fabius C Who’s Helping Whom? Examination of Care Arrangements for Racially and Ethnically Diverse People Living with Dementia in the Community. J. Appl. Gerontol. 2022, 41, 2589–2593. 35960528 10.1177/07334648221120247 PMC10348595

[18] Freedman VA; Agree EM; Seltzer JA; Birditt KS; Fingerman KL; Friedman EM; Lin I-F; Margolis R; Park SS; Patterson SE; The Changing Demography of Late-Life Family Caregiving: A Research Agenda to Understand Future Care Networks for an Aging US Population. Gerontologist 2024, 64, gnad036. 36999951 10.1093/geront/gnad036 PMC10825830

[19] Martin JA; Hamilton BE; Osterman MJK Births in the United States, 2013. NCHS Data Brief. 2014, 175, 1–8. 25483923

[20] McGarry KM Caring for the Elderly: The Role of Adult Children. In Inquiries in the Economics of Aging; University of Chicago Press: Chicago, IL, USA, 1998; pp. 133–166.

[21] Soldo BJ; Wolf DA; Agree EM Family, Households, and Care Arrangements of Frail Older Women: A Structural Analysis. J. Gerontol. 1990, 45, S238–S249. 2229950 10.1093/geronj/45.6.s238

[22] Spitze G; Logan J Sons, daughters, and Intergenerational Social Support. J. Marriage Fam. 1990, 52, 420–430.

[23] Stone R; Cafferata GL; Sangl J Caregivers of the Frail Elderly: A National Profile. Gerontologist 1987, 27, 616–626. 2960595 10.1093/geront/27.5.616

[24] Szinovacz ME; Davey A Changes in Adult Children’s Participation in Parent Care. Ageing Soc. 2013, 33, 667–697.

[25] Spillman BC; Freedman VA; Kasper JD; Wolff JL Change Over Time in Caregiving Networks for Older Adults With and Without Dementia. J. Gerontol. Ser. B 2020, 75, 1563–1572. 10.1093/geronb/gbz065 PMC7424285 31102533

[26] Wolff JL; Kasper JD Caregivers of Frail Elders: Updating a National Profile. Gerontologist 2006, 46, 344–356. 16731873 10.1093/geront/46.3.344

[27] Coleman M; Ganong L Changing Families, Changing Responsibilities: Family Obligations Following Divorce and Remarriage; Psychology Press: London, UK, 1999.

[28] Lin IF Consequences of Parental Divorce for Adult Children’s Support of Their Frail Parents. J. Marriage Fam. 2008, 70, 113–128.

[29] Arias E Changes in Life Expectancy by Race and Hispanic Origin in the United States, 2013–2014. NCHS Data Brief 2016, 244, 1–8. 27111295

[30] Baldini M; Mazzaferro C; Morciano M Assessing the Implications of Long Term Care Policies in Italy: A Microsimulation Approach. Università degli Studi di Modena e Reggio Emilia, Dipartimento di Economia Politica. Politica Econ. 2008, 24, 47–72.

[31] Cardoso T; Oliveira MD; Barbosa-Póvoa A; Nickel S Modeling the Demand for Long-Term Care Services Under Uncertain Information. Health Care Manag. Sci. 2012, 15, 385–412. 22782558 10.1007/s10729-012-9204-0

[32] Eggink E; Woittiez I; Ras M Forecasting the Use of Elderly Care: A Static Micro-Simulation Model. Eur. J. Health Econ. 2015, 17, 681–691. 26248823 10.1007/s10198-015-0714-9

[33] Laditka SB; Laditka JN Effects of Improved Morbidity Rates on Active Life Expectancy and Eligibility for Long-Term Care Services. J. Appl. Gerontol. 2001, 20, 39–56.

[34] Lymer S; Brown L; Harding A; Yap M Predicting the Need for Aged Care Services at the Small Area Level: The CAREMOD Spatial Microsimulation Model. Int. J. Microsimulation 2009, 2, 27–42.

[35] Spetz J; Trupin L; Bates T; Coffman JM Future Demand for Long-Term Care Workers Will be Influenced by Demographic and Utilization Changes. Health Aff. 2015, 34, 936–945. 10.1377/hlthaff.2015.0005 26056198

[36] Wittenberg R; Comas-Herrera A; King D; Malley J; Pickard L; Darton R Future Demand for Long-Term Care, 2002 to 2041: Projections of Demand for Long-Term Care for Older People in England. 2006. Available online: https://kar.kent.ac.uk/88828/1/dp2514.pdf (accessed on 4 February 2025).

[37] US Dept of Health and Human Services. The Future Supply of Long Term Care Workers in Relation to the Aging Baby-Boom Generation 2003; Department of Health and Human Services: Washington, DC, USA, 2003. Available online: https://aspe.hhs.gov/sites/default/files/private/pdf/72961/ltcwork.pdf (accessed on 4 February 2025).

[38] Fukawa T Household Projection and Its Application to Health/Long-Term Care Expenditures in Japan Using INAHSIM-II. Soc. Sci. Comput. Rev. 2011, 29, 52–66.

[39] Johnson RW; Toohey D; Wiener JM Meeting the Long-Term Care Needs of the Baby Boomers: How Changing Families Will Affect Paid Helpers and Institutions; Urban Institute Project TR: Washington, DC, USA, 2007.

[40] Pickard L; Wittenberg R; Comas-Herrera A; King D; Malley J Care by Spouses, Care by Children: Projections of Informal Care for Older People in England to 2031. Soc. Policy Soc. 2007, 6, 353–366.

[41] Verdery AM; Margolis R Projections of White and Black Older Adults Without Living Kin in the United States, 2015 to 2060. Proc. Natl. Acad. Sci. USA 2017, 114, 11109–11114. 28973934 10.1073/pnas.1710341114 PMC5651770

[42] Feng K; Song X; Caswell H Kinship and Care: Racial Disparities in Potential Dementia Caregiving in the US from 2000 to 2060. J. Gerontol. Ser. A: Biol. Sci. Med. Sci. 2024, 79, S32–S41. 38642100 10.1093/gerona/glae106 PMC11542221

[43] Hammel EA The SOCSIM Demographic-Sociological Microsimulation Program: Operating Manual; Institute of International Studies, University of California: Berkeley, CA, USA, 1976.

[44] Wachter KW Kinship Resources for the Elderly. Philos. Trans. R. Soc. Lond. Ser. B Biol. Sci. 1997, 352, 1811–1817. 9460065 10.1098/rstb.1997.0166 PMC1692136

[45] Rutter CM; Edochie I; Friedman EM; Slaughter ME; Weden MM A Simple Method for Simulating Dementia Onset and Death Within an Existing Demographic Model. Med. Decis. Mak. 2022, 42, 43–50. 10.1177/0272989X211016810 PMC8633039 34120512

[46] Margolis R; Verdery AM A Cohort Perspective on the Demography of Grandparenthood: Past, Present, and Future Changes in Race And Sex Disparities in the United States. Demography 2019, 56, 1495–1518. 31270779 10.1007/s13524-019-00795-1 PMC6667684

[47] Health and Retirement Study, Public Use Dataset; Produced and Distributed by the University of Michigan with Funding from the National Institute on Aging (grant numbers NIA U01AG009740 and NIA R01AG073289): Ann Arbor, MI, USA, 2016.

[48] Koller D; Bynum JP Dementia in the USA: State Variation in Prevalence. J. Public Health 2015, 37, 597–604. 10.1093/pubmed/fdu080 PMC6283394 25330771

[49] Mayeda ER; Glymour MM; Quesenberry CP; Johnson JK; Pérez-Stable E; Whitmer RA Survival After Dementia Diagnosis in Five Racial/Ethnic Groups. Alzheimers Dement. 2017, 13, 761–769. 28174069 10.1016/j.jalz.2016.12.008 PMC5496783

[50] Miller KE; Hart JL; Rosania MU; Coe NB Youth Caregivers of Adults in the United States: Prevalence and the Association Between Caregiving and Education. Demography 2024, 61, 829–847. 38785364 10.1215/00703370-11383976 PMC11539003

[51] Prudencio G; Young H Caregiving in the US 2020: What Does the Latest Edition of this Survey Tell US About Their Contributions and Needs? Innov. Aging 2020, 4, 681.

[52] Lee S; Colditz GA; Berkman LF; Kawachi I Caregiving and risk of coronary heart disease in US women: A prospective study. Am. J. Prev. Med. 2003, 24, 113–119. 12568816 10.1016/s0749-3797(02)00582-2

[53] Roth DL; Perkins M; Wadley VG; Temple EM; Haley WE Family Caregiving and Emotional Strain: Associations with Quality of Life in a Large National Sample of Middle-Aged and Older Adults. Qual. Life Res. 2009, 18, 679–688. 19421895 10.1007/s11136-009-9482-2 PMC2855243

[54] Schulz R; Beach SR Caregiving as a Risk Factor for Mortality: The Caregiver Health Effects Study. Jama 1999, 282, 2215–2219. 10605972 10.1001/jama.282.23.2215

[55] Fahle S; McGarry K How Caregiving for Parents Reduces Women’s Employment. Overtime Am. Aging Workforce Future Work. Longer 2022, 213.

[56] Lee Y; Tang F; Kim KH; Albert SM The Vicious Cycle of Parental Caregiving and Financial Well-Being: A Longitudinal Study of Women. J. Gerontol. Ser. B Psychol. Sci. Soc. Sci. 2015, 70, 425–431. 24488255 10.1093/geronb/gbu001

[57] Smith L; Morton D; van Rooyen D Family Dynamics in Dementia Care: A Phenomenological Exploration of the Experiences of Family Caregivers of Relatives with Dementia. J. Psychiatr. Ment. Health Nurs. 2022, 29, 861–872. 35088516 10.1111/jpm.12822

[58] Szinovacz ME Caring for a Demented Relative at Home: Effects on Parent–Adolescent Relationships and Family Dynamics. J. Aging Stud. 2003, 17, 445–472.

[59] Friedman EM; Kennedy DP Typologies of Dementia Caregiver Support Networks: A Pilot Study. Gerontologist 2021, 61, 1221–1230. 33585929 10.1093/geront/gnab013 PMC8599268

