# Supplementary material for: Projected Demographic Trends in the Likelihood of Having or Becoming a Dementia Family Caregiver in the U.S. Through 2060
Source: Populations (Basel). Author manuscript; Available in PMC 2026 Feb 19. (PMC12915897; doi:10.3390/populations1020010)
Supplement: Supplementary Materials [file NIHMS2131966-supplement-Supplementary_Materials.pdf]

Supplementary Materials

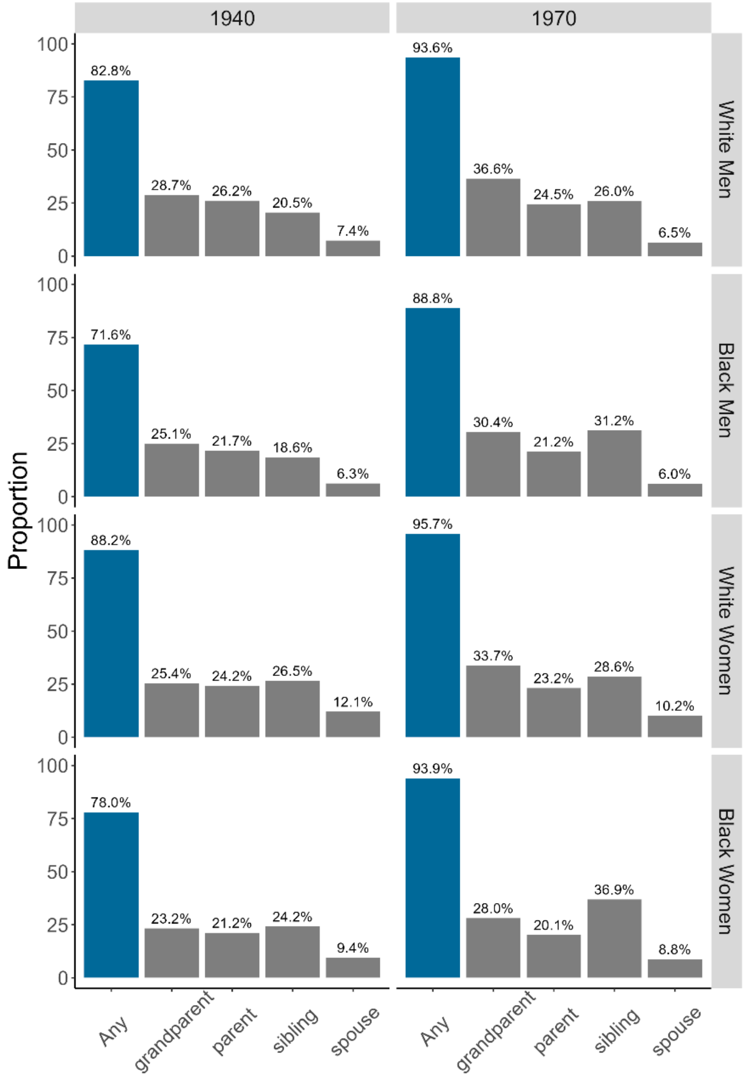

Figure S1. Fraction of the population from the 1940 and 1970 birth cohorts with relatives living with dementia by relative type, race, and gender.
